# Hard tick (Acari: Ixodidae) species of livestock and their seasonal activity in Boyer-Ahmad and Dena cities of Kohgiluyeh and Boyer-Ahmad Province, Southwest of Iran

**DOI:** 10.14202/vetworld.2018.1357-1363

**Published:** 2018-09-30

**Authors:** Zohreh Fatemian, Aref Salehzadeh, Mohammad Mehdi Sedaghat, Zakieh Telmadarraiy, Ahmad Ali Hanafi-Bojd, Amir Hosein Zahirnia

**Affiliations:** 1 Department of Medical Entomology and Vector Control, School of Medicine, Hamadan University of Medical Sciences, Hamadan, Iran; 2 Department of Medical Entomology and Vector Control, School of Public Health, Tehran University of Medical Sciences, Tehran, Iran

**Keywords:** domestic ruminants, fauna, Ixodidae ticks, seasonal activity, tick-borne diseases

## Abstract

**Aim:**

The present study was carried out to identify the Ixodidae ticks fauna of livestock and their seasonal activity in the cities of Boyer-Ahmad and Dena of Kohgiluyeh Province, south-west of Iran.

**Materials and Methods:**

Hard ticks from sheep, goats, and cattle were collected manually, stored in 70% ethanol, and identified using morphological characters.

**Results:**

During the study, a total of 1273 hard ticks from four genera, including *Rhipicephalus*, *Hyalomma*, *Dermacentor*, and *Haemaphysalis*, were collected. *Rhipicephalus sanguineus* (s.l.) had the highest frequencies in both cities with 62.08 and 62.88% of collected specimens, followed by *Hyalomma scupense* with 14.36 and 13.54% in Boyer-Ahmad and Dena, respectively. Furthermore, *Hyalomma marginatum* with only one sample or 0.12% of collected ticks showed the lowest frequencies in the studied areas. *Dermacentor marginatus* with three samples or 0.37% was recorded only in Boyer-Ahmad, and *Haemaphysalis sulcata* with two samples or 0.43% was recorded only in Dena. In both cities, sheep were the most infested ruminant, and the ears in sheep and goats were the most affected areas. The highest activity was observed in spring, and the lowest activity was observed in winter and autumn.

**Conclusion:**

The results of the present study showed that *Hyalomma* and *Rhipicephalus* genera were the most widespread genera in the study areas. Regarding the importance of genera, such as *Rhipicephalus*, *Hyalomma*, and *Haemaphysali*s, in transmitting disease agents and the location of Kohgiloyeh and Boyer-Ahmad Province in the routes of migrant birds, further studies are required to elucidate their exact roles in human and livestock health in these areas.

## Introduction

Ticks are blood-feeding obligatory ectoparasites, particularly on wild animals [1]. They are important vectors of diseases agent affecting livestock, human, and other vertebrates. About 10% of the approximately 900 known species of ticks are responsible for the transmission of numerous microorganisms among domestic animals and human [2]. These ectoparasites can transmit a variety of diseases such as Crimean-Congo hemorrhagic fever [3], tularemia, endemic relapsing fever, Lyme disease, anaplasmosis, and rickettsiosis [4]. Some tick-borne pathogens do not only threaten animal lives but also may put human health in danger. In addition to transmitting different diseases through biological or mechanical ways, ticks also can have a negative impact on human health through serious annoyance, dermatitis, fatigue, and malnutrition which can be induced by nutrition-based behaviors [5-7]. Tick paralysis in human and animals [8], as a result of the secretion of toxic substances in their saliva, is another tick-borne problem. In comparison with other blood-feeding arthropods, usually hard ticks feed for extended periods of time [4,9], which, in turn, increases the chances of transmission of diseases [10]. It is believed that pathogens transmitted by ticks are responsible for more than 100,000 cases of human disease conditions throughout the world [11] and despite the use of antiparasitic chemicals to combat the parasite, due to resistance, the control of ticks has raised concern in public and health authorities [12].

Studies on ticks in Iran were first conducted by Delpy, thereafter, Abbasian-Lintzen between 1960 and 1961 and Mazloum during 1968 and 1971 collected a list of maturated ticks from livestock [13-15]. One of the most comprehensive studies on tick’s species was conducted by Mazloum [14], who showed the geographical distribution, the hosts, active seasons, and their dispersion in different regions of the country. Hoogstraal and Valdes [16] studied the hard ticks feeding on sheep and wild goat. Nabian *et al*. [17], in their study, reported a list of tick’s species on domestic animals in Azerbaijan Saharqi. Salari-Lak *et al*. [18] also reported a list of hard ticks and soft tick’s species in Azerbaijan Qarbi. Further, Rahbari *et al*. [19] published primary reports on the distribution of various species of ticks in livestock of four geographical areas of Iran. An investigation of fauna, geographic distribution, and seasonal activity of hard ticks in Sari city resulted in collection of six species belonging to six genera of hard ticks including *Rhipicephalus bursa*, *Hyalomma detritum*, *Boophilus annulatus*, *Haemaphysalis punctata*, *Ixodes ricinus*, and *Dermacentor marginatus* [20]. Riabi and Atarodi [21] identified the fauna of hard ticks in domestic animals in the southern part of Khorasan Razavi Province and then compared them with other areas of this Province.

The majority of the domestic ruminants of Iran exist in Kohgiluyeh and Boyer-Ahmad Province. Therefore, this Province is the most important animal husbandry center in the country and that relocating them between the tropical and the cold regions (due to the seasonal changes of ambient temperature) may cause the distribution of diseases by tick infestation. Hence, this study was designed to determine the status of the diversity of ixodid ticks, seasonal distribution, topology, and their hosts which are very important in monitoring any programs to combat ectoparasites of ruminants and related diseases.

## Materials and Methods

### Ethical approval

This study obtained ethical clearance from the Ethics Committee of Hamadan University of Medical Sciences.

### Study areas

Kohgiluyeh and Boyer-Ahmad is located in the western part of Iran between latitude 30°9´ and 31°32´ N and longitude 49°57´ and 50°42´E. This Province with an area of about 16,249 km^2^ is a mountainous land and divided geographically into two large areas of the cold and tropical regions with average rainfall of 600-800 mm and 300-500 mm, respectively. The warmest months are July and August and the coldest months are January and December with average temperatures of 25-27°C and 4-5°C, respectively. The maximum and minimum registered temperature is 40 and −25°C in July and February, respectively. Dena is the tallest mountain in the Zagros Range, with more than 40 peaks higher than 4000 m and the highest peak with an elevation of 4409 m above sea level. Kohgiluyeh and Boyer-Ahmad is one of the southern Provinces of Iran. It is in the neighborhood with five Provinces of the country, from the east with Isfahan and Fars Provinces, from the south with Bushehr Province, from the west with the Khuzestan, and finally from the north with Chaharmahal and Bakhtiari Province. Agriculture and animal husbandry are the major occupations of the villagers of Boyer-Ahmad and Dena cities. Cattle are mainly kept and raised in both traditional and semi-industrial forms in animal husbandries and villages. Kohgiluyeh and Boyer-Ahmad can be divided into two: tropical and the cold regions.

### Ticks collection and identification

Ticks were collected manually from all different climate zones including plains and mountains once a month and continued for 12 months from winter of 2015 until the fall of 2016. The number of sampling was commensurate with the extent and situation of the study areas. Thus, 19 sampling sites (12 sites in Boyer-Ahmad and 7 sites in Dena city) were selected, and 600 ruminants were checked for tick infestation. Due to the dominance of mountainous area in Dena (80% mountainous and 20% plains), most sites were selected in these areas (4 spots on the mountain and 2 spots on the plains). Furthermore, Boyer-Ahmad is dominated by plain areas, and only 40% of this region is situated in mountainous areas, so the selected numbers of places in the plains were more than places in the mountains (3 spots in the plains and 2 spots in the mountains). Sampling was done on all parts of the body of livestock (cattle, sheep, and goats) and the sample size was determined based on a previous study [22]. Two important criteria in the determination of sampling sites were the geographical direction and topographical status. After restraining animals, the different areas of their bodies (head and neck, under the tail, around the anus and perineum, groin, ears, and breast) were systematically checked for the presence of ticks. To remove the ticks, cotton soaked in alcohol or chloroform was put on each tick and, thereafter, the ticks were removed using forceps. All samplings were done with care not to harm the ticks. Subsequently, the ticks were placed into tubes or cans containing 70% ethyl alcohol and 5% glycerin. The necessary information were written on all the tubes or cans including date of catching, name of the village, name of the owner of the animal, type of the animal, age, gender, and code of the livestock. After the samples were transferred to the laboratory, genus and species were diagnosed under stereomicroscope on the basis of morphological character and were compared with the characteristics presented in valid taxonomic keys [23-26] and species of the standard collection.

### Statistical analysis

Data were analyzed using Statistical Package for the Social Sciences, (SPSS 20 [IBM, USA]).

## Results

During the period of the study, a total of 1273 ticks (458 ticks in Dena and 815 ticks in Boyer-Ahmad) were collected. About 68.1% (555) of the specimens were from sheep, 22.33% (182) from goat, and 9.57% (78) from cattle in Boyer-Ahmad. Furthermore, in Dena, 52.18% (239) of specimens were collected from sheep, 37.77% (173) from goat, and 10.05% (46) from cattle. The rate of infested ruminants in the spring was more than the other seasons, and the least value was observed in winter. Tables-1 and 2 show the seasonal abundance of the hard ticks in Dena and Boyer-Ahmad cities, respectively. In Boyer-Ahmad, spring was the most active season, and 67.73% of ticks were collected during this season, but only 1.1% of ticks were collected during winter. Furthermore, in Dena, 55.24% of ticks were collected in the spring, and only 6.11% of them were collected in the autumn. *Rhipicephalus*, *Hylomma*, and *Dermacentor* were the three genera of ixodid ticks, which were identified in Boyer-Ahmad including *Rhipicephalus sanguineus* (s.l.) (62.08%), *R. bursa* (12.52%), *Rhipicephalus turanicus* (1.72%), *Rhipicephalus nymph* (0.12%), *Hyalomma asiaticum* (0.37%), *H. detritum* (0.12%), *Hylomma excavatum* (1.1%), *Hyalomma marginatum* (0.12%), *Hyalomma scupense* (14.36%), *Hylomma* sp. (1.35%), *Hylomma nymph* (5.77%), and *D. marginatus* (0.37%). Furthermore, three genera of ixodid ticks (*Rhipicephalus, Hylomma*, and *Haemaphysalis*) were identified in Dena, which includes *R*. *sanguineus* (s.l.) (62.88%), *R. bursa* (12.45%), *R. turanicus* (1.32%), *R. nymph* (0/43%), *H. scupense* (13.54%), *H. marginatum* (0.22%), *H. asiaticum* (0.43%), *H. nymph* (6.77%), *Hylomma* sp. (1.53%), and *Haemaphysalis sulcata* (0.43%). *R. sanguineus* (s.l.) was the most abundant hard tick in the studied cities.

**Table-1:** Seasonal abundance of hard ticks in the Boyer-Ahmad city.

Species	n (%)	Season									

		Spring		Summer		Autumn		Winter		Total	
				
		Female	Male	Female	Male	Female	Male	Female	Male	Female	Male
*R. sanguineus*	506 (62.08)	173	244	17	37	9	17	5	4	204	302
*R. bursa*	102 (12.52)	62	37	1	2	0	0	0	0	63	39
*R. turanicus*	14 (1.72)	0	14	0	0	0	0	0	0	0	14
R. nymph*	1 (0.12)	-	-	-	-	-	-	-	-		-
*H. asiaticum*	3 (0.37)	0	2	0	0	0	1	0	0	0	3
*H. detritum*	1 (0.12)	1	0	0	0	0	0	0	0	1	0
*H. excavatum*	9 (1.1)	0	1	0	2	3	3	0	0	3	6
*H. marginatum*	1 (0.12)	0	0	0	0	0	1	0	0	0	1
*H. scupense*	117 (14.36)	0	12	37	58	5	5	0	0	42	75
*Hyalomma* spp.	11 (1.35)	3	0	1	0	7	0	0	0	11	0
H. nymph*	47 (5.77)	-	-	-	-	-	-	-	-	-	-
*D. marginatus*	3 (0.37)	2	1	0	0	0	0	0	0	2	1
Total	815 (100)	241	311	54	99	24	27	5	4	326	441

* Sex in the nymphs is unknown. *R. sanguineus=Rhipicephalus sanguineus*, *R. bursa=Rhipicephalus bursa*, *R. turanicus=Rhipicephalus turanicus, R.* Nymph=*Rhipicephalus* nymph, *H. asiaticum=Hyalomma asiaticum*, *H. detritum=Hyalomma detritum, H. excavatum=Hylomma excavatum, H. marginatum=Hyalomma marginatum, H. scupense=Hyalomma scupense, H.* nymph=*Hylomma* nymph*, D. marginatus=Dermacentor marginatus*

**Table-2:** Seasonal abundance of hard ticks in the Dena city.

Species	n (%)	Season									

		Spring		Summer		Autumn		Winter		Total	
				
		Female	Male	Female	Male	Female	Male	Female	Male	Female	Male
*R. sanguineus*	288 (62.88)	93	106	12	30	8	10	21	8	134	154
*R. bursa*	57 (12.45)	40	11	2	1	0	0	0	3	42	15
*R. turanicus*	6 (1.32)	0	1	0	0	0	0	0	5	0	6
R. nymph*	2 (0.43)	-	-	-	-	-	-	-	-	-	-
*H. scupense*	62 (13/54)	0	1	12	47	1	1	0	0	13	49
*H. marginatum*	1 (0.22)	0	0	0	0	0	1	0	0	0	1
*H. asiaticum*	2 (0.43)	0	0	0	0	0	2	0	0	0	2
*Hyalomma* spp.	7 (1.53)	0	0	1	2	4	0	0	0	5	2
H. nymph*	31 (6.77)	0	0	0	0	0	0	0	0	0	0
*H. sulcata*	2 (0.43)	1	0	0	0	0	0	0	1	1	1
Total	458 (100)	134	119	27	80	13	14	21	17	195	230

* Sex of nymphs is unknown. *R. sanguineus=Rhipicephalus sanguineus*, *R. bursa=Rhipicephalus bursa*, *R. turanicus=Rhipicephalus turanicus, R. nymph=Rhipicephalus nymph, H. scupense=Hyalomma scupense, H. marginatum=Hyalomma marginatum, H. asiaticum=Hyalomma asiaticum*, *H. nymph=Hylomma nymph, H. sulcate=Haemaphysalis sulcata*

**Figure-1:**
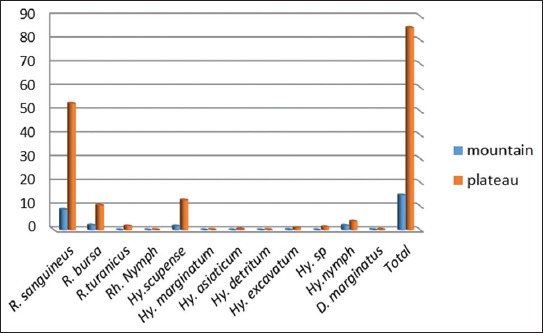
Topological preference of hard ticks collected in the Boyer-Ahmad city.

**Figure-2:**
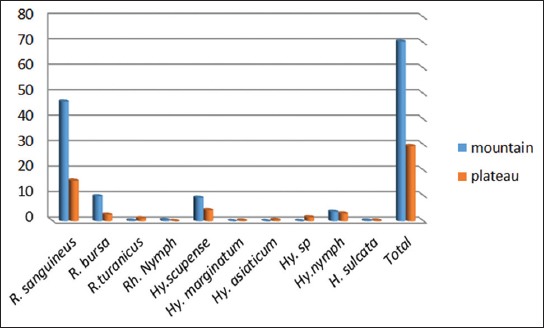
Topological preference of hard ticks collected in the Dena city.

Figures-1 and 2 show the topological preference of hard ticks in both cities. In Boyer-Ahmad, 85.28% of ticks were collected from the plains and 14.72% from the mountainous areas, whereas in Dena city, only 29.33% of ticks were collected from the plains and 70.67% of samples were obtained from the mountainous area. The genus *Rhipicephalus* was the most abundant collected from sheep, whereas *Hyalomma* was the most abundant genus collected from cattle (Tables-3 and 4). *H. asiaticum*, *H. excavatum*, *H. marginatum*, and *R. turanicus* only were found on sheep.

**Table-3:** The host diversity of hard tick species in the Dena city.

Species	Host		

	Cattle	Sheep	Goat
*R. sanguineus*	7	154	127
*R. bursa*	3	26	28
*R. turanicus*	0	0	6
*R.* nymph	2	0	0
*H. scupense*	27	28	7
*H. marginatum*	0	1	0
*H. asiaticum*	0	2	0
*Hyalomma* spp.	2	5	0
*H.* nymph*	5	22	4
*H. sulcata*	0	1	1
Total	46	239	173

* Sex of Nymphs is unknown. *R. sanguineus=Rhipicephalus sanguineus*, *R. bursa=Rhipicephalus bursa*, *R. turanicus=Rhipicephalus turanicus, R.* nymph=*Rhipicephalus nymph, H. scupense=Hyalomma scupense, H. marginatum=Hyalomma marginatum, H. asiaticum=Hyalomma asiaticum*, *H.* nymph*=Hylomma* nymph, *H. sulcate=Haemaphysalis sulcata*

**Table-4:** The host diversity of hard tick species in Boyer-Ahmad city.

Species	Host		

	Cattle	Sheep	Goat
*R. sanguineus*	38	314	154
*R. bursa*	0	89	13
*R. turanicus*	0	14	0
*R.* nymph	0	1	0
*H. asiaticum*	0	3	0
*H. detritum*	0		1
*H. excavatum*	0	9	0
*H. marginatum*	0	1	0
*H. scupense*	40	70	7
*Hyalomma* spp.	0	11	0
*H.* nymph*	0	41	6
*D. marginatus*	0	2	1
Total	78	555	182

* Sex of nymphs is unknown. *R. sanguineus=Rhipicephalus sanguineus*, *R. bursa=Rhipicephalus bursa*, *R. turanicus=Rhipicephalus turanicus*, *R.*nymph=*Rhipicephalus* nymph, *H. asiaticum=Hyalomma asiaticum*, *H. detritum=Hyalomma detritum*, *H. excavatum=Hylomma excavatum*, *H. marginatum=Hyalomma marginatum*, *H. scupense=Hyalomma scupense*, *H.* nymph*=Hylomma nymph*, *D. marginatus=Dermacentor marginatus*

**Figure-3:**
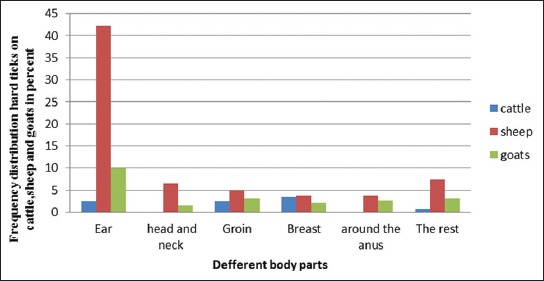
Body-site distribution of hard ticks collected from cattle, sheep, and goats in the city of Boyer-Ahmad.

**Figure-4:**
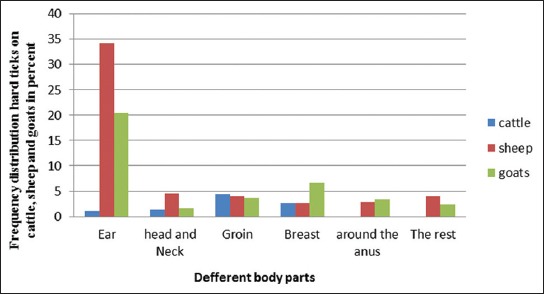
Body-site distribution of hard ticks collected from cattle, sheep, and goats in the city of Dena.

Figures-3 and 4 show the body-site distribution of ticks collected from the ruminants. As shown in the figures, the greatest number of ticks collected from sheep and goats was from the regions of their ears, whereas most of the ticks collected from cattle were from their groin and breast regions.

## Discussion

The present study was conducted throughout a whole year, with an attempt to determine comprehensive information about fauna and geographical distribution of various species of ticks in different seasons, as well as host preference and body-site distribution in Boyer-Ahmad and Dena cities.

A total of 1273 hard ticks from cattle, sheep, and goats, belonging to four genera (*Rhipicephalus*, *Hylomma*, *Haemaphysalis*, and *Dermacentor*), were collected. The reported genera in this research have been considered as very important hard ticks from the medical and veterinary perspective in Iran [4,11,19].

*R. sanguineus* (s.l.), which is also known as the brown dog tick, was most abundant and widespread tick species throughout the studied areas. The similar result had also been reported in other studies in Iran [6,16,25,26] and throughout the world [27]. Due to its endophilous nature, this species is adapted to live within human dwellings and is active throughout the year. Some reports suggested that in warm climate or regions with longer summer, *R. sanguineus* (s.l.) readily attach to man [28]. This, in turn, increases the risk of transmission of pathogens such as *Rickettsia conorii* to human and *Ehrlichia canis* or *Babesia canis vogeli* to dogs [29]. *R. sanguineus* (s.l.), which has been considered as a species complex and parasitizes a wide range of vertebrates [30], has also been suggested as suspected vectors of sheep anaplasmosis in the central part of Iran [31].

The results of the present study showed that sheep and goats were infested to a greater extent than cattle (36.76, 22.15, and 3.53% infestation, respectively). These results are in line with those reported in studies by Abbasian-Lintzen [13] and Mazlum [14] and a similar study in Golestan Province, north of Iran [16]. Adult *R. bursa* has been reported as a vector of *Babesia ovis* [32]. This species had a similar frequency in both cities, confirming the adaptability of this parasite to different geographical conditions and hosts [33]. The distribution of *R. bursa* by livestock and other hosts can increase the chances of disease transmission. *R. turanicus* is distributed mainly in Africa and Asia and a large part of the Mediterranean to India and China [34]. Although in a study conducted by Razmi *et al*. [35] in the North and South of Khorasan Razavi, *R. turanicus* was reported as dominant species, and the frequencies of this species in Boyer-Ahmad and Dena were found to be only 1.72% and 1.32% of the collected ixodid ticks, respectively.

In addition to mammals, birds are potential hosts of ixodid ticks [36]. As a result of the to and fro movement of wild bird from Iran, it is probable that some species of *Hyalomma* or *Haemaphysalis*, which are carried by migratory bird [37,38], can transmit some disease agents between Iran and other countries. Ticks of genus *Haemaphysalis* are found in all weather conditions and geographical areas but are more prevalent in wet weather [18]. In the present study, *H. sulcata* was collected only from Dena city. Since Dena is situated in higher altitude, the difference in weather and other environmental factors may justify the absence of this species from Boyer-Ahmad. *D. marginatus* the only known species of the genus *Dermacentor* was collected from sheep and goat of Boyer-Ahmad. These species are distributed in many parts of Western Europe to Western Kazakhstan, especially in the lowlands and deciduous forested areas [27]. It has also been reported in different parts of Iran by some researchers [26,39]. *D. marginatus* and some other species of *Dermacentor* are known vectors of *Rickettsia slovaca* and *Rickettsia raoultii* [40].

In the present study, the number of collected ticks was higher in spring and summer which indicate the dependence of ticks to ambient temperature. The importance of environmental conditions such as enough rainfall and heat on the activity of ticks have also been mentioned by other researchers [27]. The region has an appropriate climate, vegetation cover, and sufficient heat suitable for completing the life cycle of ticks in warmer seasons. The study was conducted in plain and mountainous areas of Boyer-Ahmad and Dena. In Boyer-Ahmad, 85.28% of ticks were collected from the plain areas, whereas only 29.33% of the ticks were obtained from the plain areas of Dena. On the contrary, 70.67% of ticks were collected from the mountainous regions Dena.

The results of the present study showed that *Hylomma* and *Rhipicephalus* genera were the most widespread genera distributed in the study areas. Regarding the role of *R. burs*a in transmitting *Babesi*a spp. in cattle, sheep, goat, horse and dog [41], and *Hylomm*a spp. in transmitting Theileria [42] and Crimean-Congo haemorrhagic fever [43], as well as the role of *Haemaphysali*s spp. in circulation of some rickettsial diseases [44], further studies are required to elucidate their exact roles in the epidemiology of human and animal diseases. Furthermore, some Iranian Provinces including Kohgiluyeh and Boyer-Ahmad are in the migration routes of birds [45], and as such, the likelihood of spreading tick-borne diseases by migratory bird to and fro Iran should be considered.

## Conclusion

The results of the present study can provide a basis for the adoption of an effective strategy for the management of hard ticks in livestock of Kohgiluyeh and Boyer-Ahmad. Due to the expanded livestock industry, appropriate situation for the development and dispersion of hard ticks, the status of tick-borne diseases and the rate of infestation of animals should be monitored routinely. Moreover, the regular and accurate dipping of livestock can be one of the most important methods to combat the mentioned ectoparasites and related diseases, especially in sheep and goats.

## Authors’ Contributions

AS, ZT and AHZ conceptualized and designed the study. ZF collected the samples. ZF, AS and ZT identified the samples. AS and ZF analyzed data and drafted the manuscript. MMS, AS, AHZ, AAH and ZT supervised the project. All authors discussed the results and implications and commented on the manuscript at all stages. All authors read and approved the final manuscript.
